# The Angular Spectrum of the Scattering Coefficient Map Reveals Subsurface Colorectal Cancer

**DOI:** 10.1038/s41598-019-39146-w

**Published:** 2019-02-28

**Authors:** Yifeng Zeng, Bin Rao, William C. Chapman, Sreyankar Nandy, Rehan Rais, Iván González, Deyali Chatterjee, Matthew Mutch, Quing Zhu

**Affiliations:** 1 Department of Biomedical Engineering, Washington University, St. Louis, MO USA; 2 Department of Surgery, Section of Colon and Rectal Surgery, Washington University School of Medicine, St. Louis, MO USA; 3 Department of Pathology and Immunology, Washington University School of Medicine, St. Louis, MO USA; 4 Department of Radiology, Washington University School of Medicine, St. Louis, MO USA

## Abstract

Colorectal cancer diagnosis currently relies on histological detection of endoluminal neoplasia in biopsy specimens. However, clinical visual endoscopy provides no quantitative subsurface cancer information. In this *ex vivo* study of nine fresh human colon specimens, we report the first use of quantified subsurface scattering coefficient maps acquired by swept-source optical coherence tomography to reveal subsurface abnormities. We generate subsurface scattering coefficient maps with a novel wavelet-based-curve-fitting method that provides significantly improved accuracy. The angular spectra of scattering coefficient maps of normal tissues exhibit a spatial feature distinct from those of abnormal tissues. An angular spectrum index to quantify the differences between the normal and abnormal tissues is derived, and its strength in revealing subsurface cancer in *ex vivo* samples is statistically analyzed. The study demonstrates that the angular spectrum of the scattering coefficient map can effectively reveal subsurface colorectal cancer and potentially provide a fast and more accurate diagnosis.

## Introduction

Cancer of the colon and rectum is the second most common malignancy diagnosed globally and represents the 4^th^ leading cause of cancer mortality. In the US, approximately 107,000 cases of colorectal cancer are diagnosed annually^1,2^. Arising from neoplastic growth on from the inner surface–or mucosal layer–of the colon, these cancers can penetrate through the deeper layers of the colon and spread to other organs. Left untreated, the disease is fatal. Detection and treatment before the cancer can penetrate the mucosal base is key; multiple studies have demonstrated the survival advantages of screening and subsequent early intervention^3,4^. Screening for colorectal malignancy is performed by flexible endoscopy, which relies on a camera for visual inspection of the mucosal lining of the colon and rectum.

However, this technique relies on visual detection of surface abnormallities, and no subsurface quantitative information is provided. Often, early malignancies can be missed^5–7^. Additionally, some colorectal cancers need to be clinically evaluated after treatment with radiation and chemotherapy. However, current endoscopy and radiographic imaging modalities cannot accurately differentiate residual scar from remaining nests of tumor. In some colorectal malignancies, this limitation is the greatest barrier to novel treatment approaches such as nonoperative management. Therefore, to improve screening and surveillance of colorectal cancers, better screening and diagnostic imaging modalities are needed.

To address this need, we investigated the feasibility of using optical coherence tomography (OCT), an established technique providing high resolution imaging^8–12^, for differentiating cancerous and normal human colon tissues. OCT has already become a standard imaging modality in ophthalmology^13,14^. The use of intravascular OCT has grown exponentially for investigating coronary atherosclerosis, and it has similar potential for monitoring therapy^15–18^. Endoscopic adaptations of OCT have also been demonstrated in the upper gastrointestinal tract, where it is used to assist with esophageal screening in the setting of Barrett’s esophagus^19–22^.

OCT’s main advantage is providing subsurface structure information in early neoplastic progression, when the colon undergoes subsurface architectural disruption that is invisible to the naked eye^23^. Previously, several research groups have explored the technical feasibility of performing endoscopic OCT imaging within mouse and human large bowel models as well as quantitative feature extraction of rodent and human bowel. Adler *et al*. found that it was possible to acquire 3-D *in vivo* OCT images in human colons^24^. Welge *et al*. demonstrated the feasibility of endoscopic Doppler OCT images in mouse models^25^. These studies have focused on the feasibility of endoscopic OCT rather than quantification. In the meantime, several research groups have developed various methods for quantifying optical images generated from rodent colons. Roy *et al*. applied 4-D elastic light-scattering fingerprints^26^ and Robles *et al*. extracted depth-resolved spectroscopic information^27^. Further, Winkler *et al*. quantified three grayscale-based features using OCT^28^ and Tang *et al*. generated scattering coefficient maps in mice colons using OCT^29^. Tang’s method provided a nice visualization of malignant mice colon tissue optical properties. Finally, Qi *et al*., Terry *et al*., Wang *et al*., and Yang *et al*. applied quantitative approaches to various human tissues. Qi *et al*. quantified *in vitro* colonic crypt morphology^30^ and Terry *et al*. quantified the nuclear diameter and density in *ex vivo* human colons^31^. More related, Wang *et al*. used spectral domain OCT to extract scattering coefficient from human colorectal polyps^32^ and Yang *et al*. calculated the scattering coefficient from human ovary specimens using swept-source OCT^33^. All these studies show statistical significance of quantified OCT features. These studies of human tissue provide the foundation for quantitative analysis of OCT A-scan images. Here, we have presented scattering maps using 3-D swept-source OCT images of human colorectal tissues and further extracted features from these scattering maps.

In this communication, for the first time, we report an *ex vivo* study to reveal subsurface abnormities in nine fresh human colon specimens using quantified subsurface scattering coefficient maps acquired by swept-source optical coherence tomography (SSOCT). We generate subsurface scattering coefficients with a novel wavelet-based-curve-fitting method with significantly improved accuracy. Scattering coefficient maps are extracted from OCT C-scans providing a visualization of tissue optical properties. A 2-D Fourier transformation generates angular spectrums from scattering coefficient maps. An angular spectrum index (ASI) is derived to quantify the differences between the normal and abnormal tissues, and its strength in revealing subsurface cancer in *ex vivo* colorectal specimens is statistically analyzed. These preliminary results demonstrate the feasibility of using quantified SS-OCT to identify early mucosal neoplasms within the human colon.

## Results

### Generating scattering coefficients with a wavelet-based-curve-fitting method

**Figure 1:**
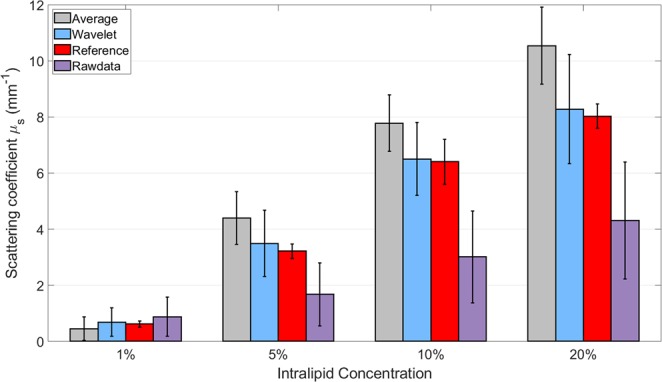
Phantom scattering coefficient measurement. The reference scattering coefficient for each group of intralipid (red bar) is compared to three alternative coefficients calculated by the Near-by-average curve fitting method (gray bar), the Wavelet-based curve fitting method (blue bar), and the Raw data curve fitting method (purple). Error bars represent the standard deviation of each method. The wavelet-based-curve-fitting method (blue bar) best correlates with the accepted standard measurement value for each of the intralipid phantoms.

Based on Beer’s law, the scattering coefficients were quantified using SSOCT A-line signals from phantoms with negligible absorption^34^ (see *Methods*). The scattering coefficients of the colorectal samples mostly fell within the range of the phantoms. Four different concentrations of intralipid of 1%, 5%, 10%, and 20% were used as liquid phantoms resulting in four measured scattering coefficients of 0.62 *mm*^−1^, 3.22 *mm*^−1^, 6.41 *mm*^−1^, and 8.03 *mm*^−1^ respectively. The measured results of the 1% and 20% intralipid were comparable to available literature data: 0.6 *mm*^−1^ for 1% intralipid^35^ and 8 *mm*^−1^ for 20% intralipid^36^. Three OCT datasets were used for quantification: data after wavelet analysis, data after nearby A-line average, and raw data. Three scattering coefficients calculated by the above three methods are compared to the measured scattering coefficient in Fig. 1. Red identifies the measured scattering coefficients which is used as reference scattering coefficient. Surrounding each red data point, scattering coefficients derived from the three methods (average, wavelet, and raw data) are displayed for the same phantom with error bars. Among all three methods, wavelet-based analysis (blue) best correlates with the accepted standard measurement value for each of the intralipid phantoms. The average method (gray) overestimates the scattering coefficients, while the raw-data method (purple) underestimates the scattering coefficients; comparisons of each method are provided in Table 1. Based on these results, we selected the wavelet-based-curve-fitting method for estimating scattering coefficients with OCT. It is worth mentioning that the wavelet multiresolution decomposition method is a well-developed speckle noise reduction method^37–42^.

**Table 1:** Comparison of scattering coefficients derived by three alternative analysis methods.

Intralipid concentration	Standard Measurement Mean *μ*_*s*_ (*mm*^−1^)	Wavelet-based Curve Fitting Mean *μ*_*s*_ (*mm*^−1^)	Error (%)	Near-by-average Curve Fitting Mean *μ*_*s*_ (*mm*^−1^)	Error (%)	Raw data Curve Fitting Mean *μ*_*s*_ (*mm*^−1^)	Error (%)
1%	0.6203	0.6902	11.2	0.4505	27.4	0.8788	41.7
5%	3.2202	3.4916	8.4	4.3978	36.6	1.6767	47.9
10%	6.4088	6.5048	1.5	7.7813	13.7	3.0173	52.9
20%	8.0320	8.2821	3.1	10.543	31.3	4.3148	46.3

### OCT images of normal and cancerous specimens

**Figure 2:**
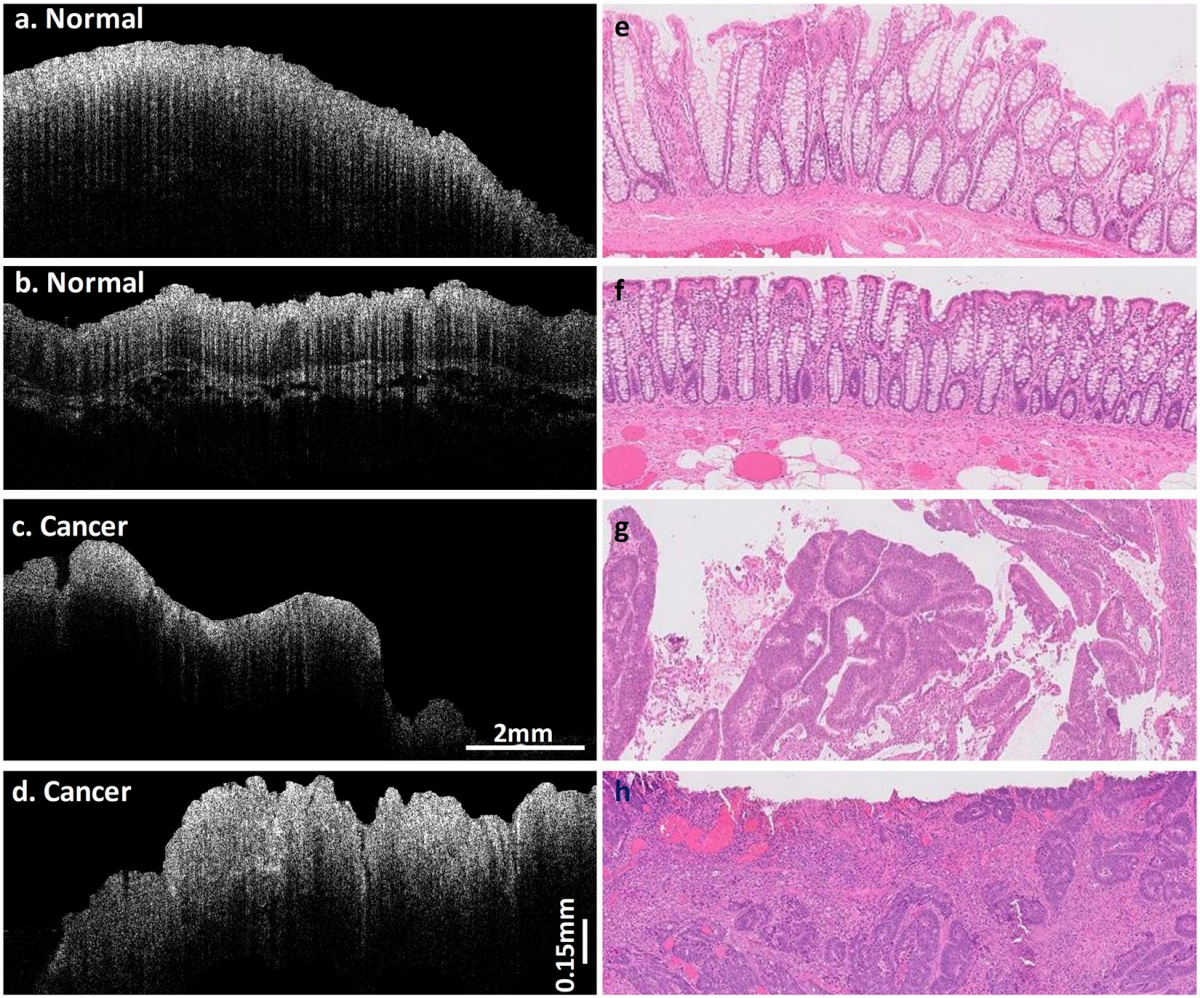
OCT images of normal and cancer colon specimens. (**a**,**b**) are typical normal tissue SS-OCT B-scan images from different patients. **e** and **f** are corresponding H&E slides. (**c**,**d**) are selected abnormal tissue SS-OCT B-scan images from the same patients as (**a**,**b**), respectively. (**g**,**h**) are H&E slides of the same malignancy.

Representative SS-OCT B-scan images of normal colon tissues, cancerous tissues, and corresponding H&E slides are shown in Fig. 2. The OCT and histologic images have the same scale and come from similar, but not identical, locations within the colon specimens. All the images are 0.75 mm wide and 10 mm long. Figures 2a,b are representative OCT B-scan images from two normal specimens. A dentate line structure, which may correlate to the presence of a regular and well-organized crypt pattern in the epithelium, is observed in these images. These serrated edges in the OCT images correspond to the surface morphology shown in their histology results in 2e and 2f.

However, the cancerous tissues present themselves differently in their OCT images, shown in Fig. 2b,d. Significant surface erosions in cancer OCT Fig. 2c,d could be attributed to cancer invasions of the surface tissue. In the corresponding histology images 2 g and 2 h, the cancerous tissue appears highly irregular compared to the normal specimens, and shows a loss of normal colonic architecture.

### Scattering coefficient maps of human colorectal specimens

**Figure 3:**
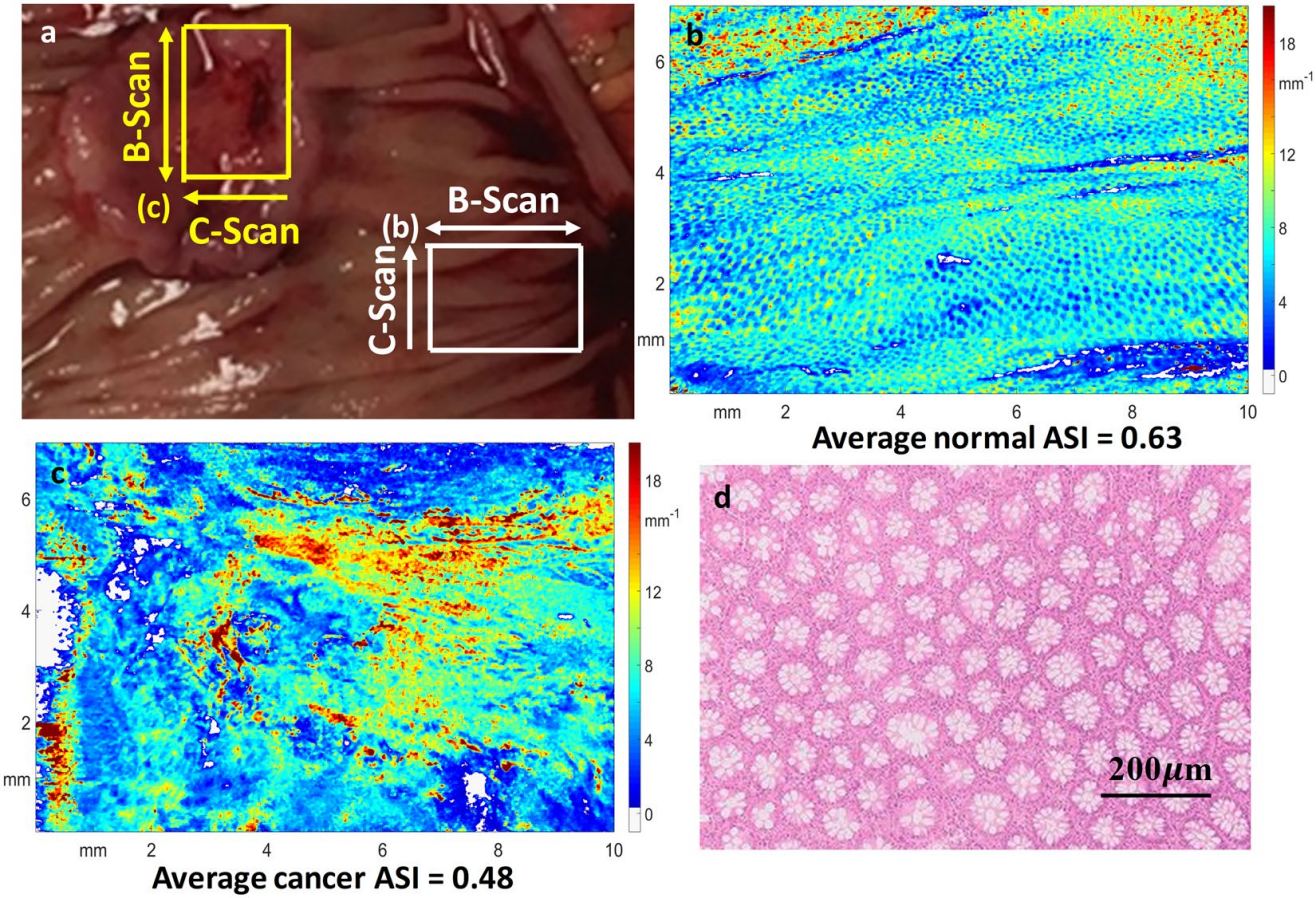
Scattering coefficient maps. (**a**) Is a photograph of the colon specimen. The white box marks the imaged normal tissue area, while the yellow box delineates the processed malignant tissue. (**b**) Is the scattering coefficient map from the normal tissue. (**c**) Is the scattering coefficient map of the cancer. (**d**) Illustrates the histological crypt structure of normal surface mucosa visualized in coronal cross section.

**Figure 4:**
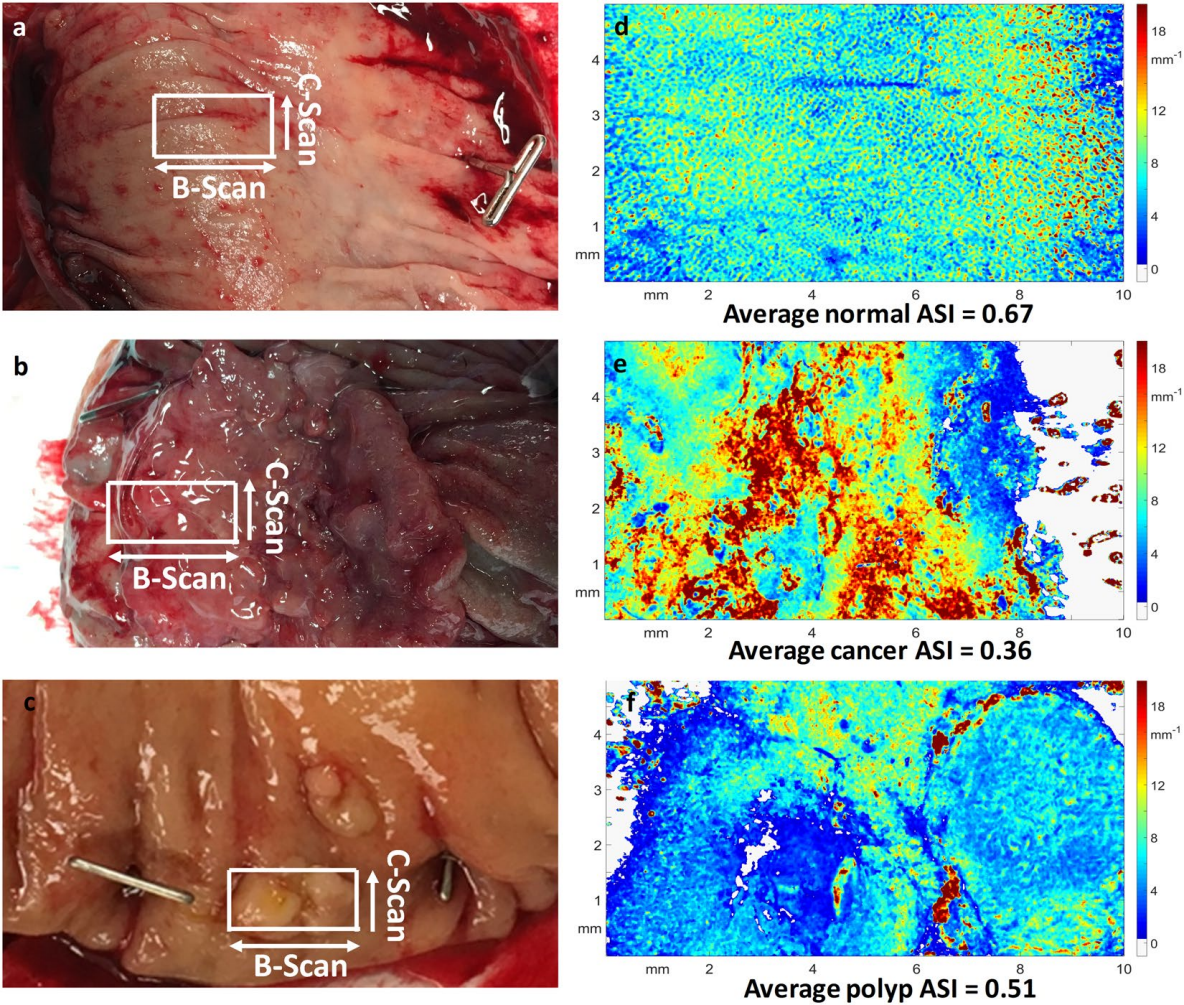
Scattering coefficient maps. (**a**,**b**) Are gross photographs of normal and cancerous regions of one colon specimen, respectively. The white block approximately shows the processed area. (**c**) Is a gross photograph of a polyp specimen, (**d**) shows the scattering map of the normal region, (**e**) is the scattering map of the cancer region, and (**f)** presents the polyp scattering map.

We performed 3-D mapping of the scattering coefficients of the epithelium layer and then derived the ASI of each map, as described in the Methods section. Figures 3 and 4 show scattering coefficient maps from colorectal specimens of three patients and one corresponding histology result. The white areas are regions that are out of focus.

The *en face* scattering coefficient maps of the normal colons (Figs 3b and 4d) contain a large area of homogenous scattering coefficients with periodic dot patterns, while the scattering coefficient map of cancer region (Figs 3c and 4e) shows a large area of heterogeneous scattering coefficients. The normal map matches very well with the histological *en face* crypt structure, which is shown in magnification in Fig. 3d. The average dot-diameter (from eight random dots) is 67.88 *μm* in the scattering map and 69.75 *μm* in the histology, a very close match. The colon samples in Fig. 4 do not have such a close histological comparison because of the pathological process. The scattering distribution relates highly to the cancer’s shape and inner structure. Since each colon cancer case can be pathologically different, there are no common features except for highly irregularity in the scattering coefficient maps of cancer tissues. Figure 4f shows the scattering coefficient map of a polyp that has been shown to be precancerous. The periodic pattern can hardly be visualized, and the map shows heterogeneity due to the abnormal growth.

**Figure 5:**
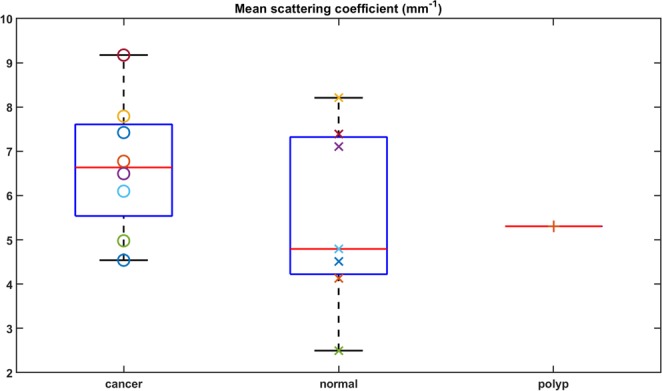
Mean Scattering Coefficients. The mean scattering coefficients for all imaged specimens are shown. The cancerous specimens’ mean scattering coefficients are ranged from 4.54 *mm*^−1^ to 9.17 *mm*^−1^. And the mean scattering coefficients for normal tissues lie between 2.50 *mm*^−1^ and 8.21 *mm*^−1^. The mean scattering coefficient for the polyp case is 5.31 *mm*^−1^.

The mean scattering coefficients for all imaged specimens are shown in Fig. 5. The cancerous specimens’ mean scattering coefficients ranged from 4.54 *mm*^−1^ to 9.17 *mm*^−1^, while the mean scattering coefficients for normal tissues lie between 2.50 *mm*^−1^ and 8.21 *mm*^−1^. The mean scattering coefficient for the polyp case is 5.31 *mm*^−1^. As we can see from Fig. 5, there is overlap between normal and cancer values due to the large variation of scattering coefficients within these tissue types. This observation of heterogeneity within the normal colonic mucosa could be due to different segments of colon imaged, variable amounts of ischemic time or the health status of the patient.

### Angular spectrum analysis and its strength in revealing subsurface cancer

**Figure 6:**
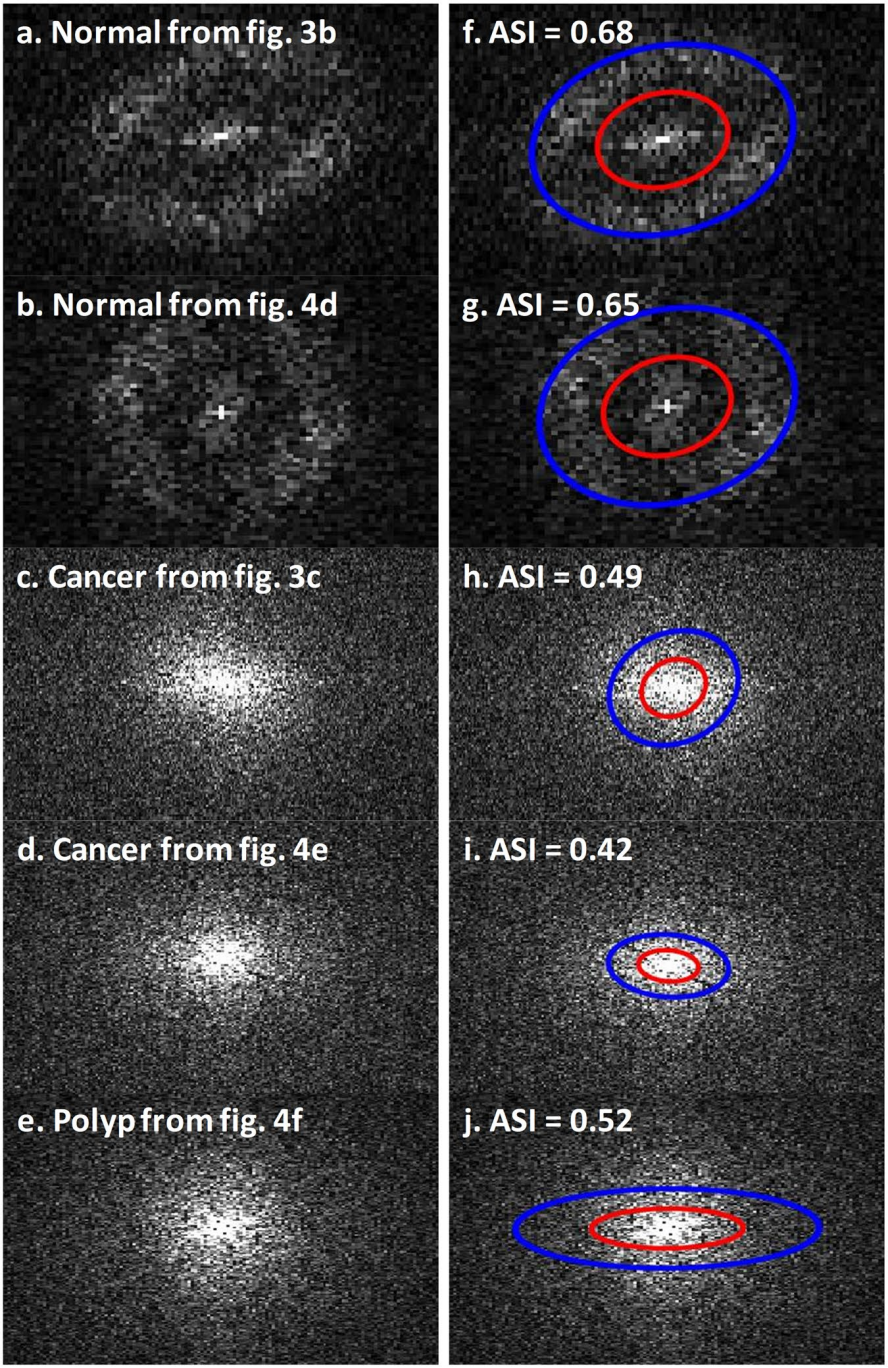
Angular spectrum images and their angular spectrum indexes quantified with a two-ellipse method.

Nine colon specimens, including eight cancerous regions, five normal regions, and one pre-malignant polyp were successfully imaged and processed. The angular spectrum was acquired by applying 2-D Fourier transform to selected regions of the scattering coefficient maps, with the results shown in the left column of Fig. 6. The right column of Fig. 6 shows ellipses on top of angular spectra for quantification of ASI according to the Methods section. Specifically, we use a Sobel edge detection method to find the area with valid signal, and then fit an ellipse using the least squares criterion (blue ellipses). Red ellipses are ellipses with quarter of the area of blue ellipses. While the blue ellipses include most of the scattering coefficient signals, the red ellipses enclose lower frequency components, corresponding to an inhomogeneous scattering coefficient distribution. Most high-frequency components, corresponding to a homogeneous and periodic scattering coefficient distribution, are outside of the red ellipse.

An angular spectrum ring between the red ellipse and blue ellipses can be observed only in the normal cases. The angular spectrum ring corresponds to clear separation between the frequency components of the homogeneous and periodic scattering coefficient distribution and the spatial frequency components of the inhomogeneous scattering coefficient distribution. From the histology result (Fig. 3d), we calculated the average spatial frequency to be 12.93 *mm*^−1^; from the angular spectrum, we derived the average spatial frequency to be 11.89 *mm*^−1^. Unfortunately, due to the pathological processing, we were not able to obtain histological results for other normal regions of imaged colon specimens. However, we estimate the range to be 9.41 *mm*^−1^ to 16.93 *mm*^−1^ based on other normal angular spectrums.

**Figure 7:**
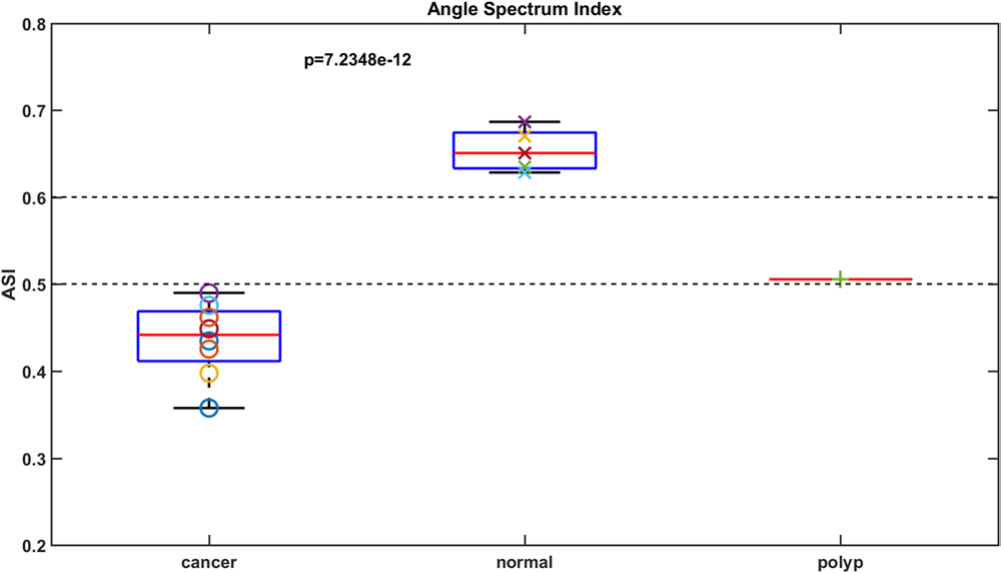
Angular spectrum index. Eight cancer tissues, five normal tissues, and one polyp are quantified. The p-value is between cancer and normal. The two dotted lines are used for visualizing the separation.

To measure the ring structure, the ASI was quantified by taking the ratio of the higher frequency components (integration of all signals in between the red and blue ellipse) to all frequency components (integration of all signals within the blue ellipse). This index separates five normal tissues from eight cancer tissues (Fig. 7, p-value <<0.001). One polyp, which is a precancerous lesion, is also shown in Fig. 7. It sits between the normal and cancerous tissues, which indicates a gradual structural change and the potential of using ASI to detect early stage colon cancer.

## Discussion

In this pilot study, we evaluated the feasibility of qualitatively and quantitatively differentiating malignancies from normal colon tissue through optical coherence tomography (OCT). Scattering coefficient maps and angular spectrum analysis were calculated from OCT images generated from known malignant and normal tissue immediately after surgical resection. Qualitatively, 3-D scattering coefficient mapping of these specimens suggested unique subsurface microscopic optical scattering patterns that appear to differentiate malignant from normal tissue. Specifically, subsurface cancers destroy the homogenous crypt pattern seen in normal tissues and create random distributions. Quantitatively, angular spectra of the scattering maps demonstrate higher frequency components in normal tissues, shown as an angular spectrum ring pattern (Fig. 6). Further ASI quantification reveals the spatial frequency range of the normal crypt pattern, which significantly varies from ASI in cancerous tissues. Some highly scattering regions may look heterogeneous in normal scattering coefficient maps due to stronger scattering, e.g., the upper right portion of Fig. 4b. We cropped and visualized this region separately and it shows a periodic pattern similar to normal regions. Moreover, we have quantified the ASI of this cropped area and it lies within normal tissue ranges. (More details in Supplementary Materials.)

Based on these findings, our system appears to differentiate organized normal colonic architecture from the irregular heterogeneous areas observed in malignant histology within this limited pilot study. Recent studies have shown that changes in crypt size and appearance are associated with the earliest forms of colorectal cancer^43^; therefore, OCT’s ability to image the mucosal architecture in real time may lead to more sensitive assessment of early malignancies and improved detection of residual malignant tissue after chemotherapy and radiation treatment.

We also hypothesize that ASI quantification may be a key, objective tissue characteristic that informs clinical decision-making when evaluating the large bowel for cancer. Several studies have already shown the potential of camera-guided endoscopic OCT. Shen *et al*. demonstrated colonoscopic OCT for detecting transmural inflammation in inflammatory bowel disease^44^, and Zagaynova *et al*. used camera-guided OCT to detect polyps^45^. These studies focused on 2-D B-scan images. To reduce the potential for inter-operator variability when interpreting scatter coefficient maps, we hypothesize that 3-D C-scan imaging with real-time ASI quantification may provide objective clinical data that augments endoscopic evaluation. OCT has also been utilized as a correlation method for tissue angiography and microcirculation detection^46–49^.

However, several technical limitations currently reduce the clinical efficacy of the specific system as described. All image post-processing is based on CPU running MATLAB. The total image post-processing time for a 5 mm by 1 cm (500 B-scans, 1000 A-lines/B-scan, and 1024 pixels/A-line) area is twelve hours on a Dell Inspiron 3650 (x64-based, Intel i5-6400 CPU @ 2.70 GHz, 8GB RAM). To produce clinically relevant results, however, image processing and ASI quantification must be achieved in significantly shorter amounts of time. Future system improvements will therefore focus on GPU implementation and algorithm optimization to improve computational speed and accuracy. Then, *in vivo* study of system performance will be undertaken in an appropriately powered study to evaluate the clinical efficacy of this promising technology.

The data presented here suggest that OCT imaging may produce qualitative and quantitative information that differentiates malignant from normal tissue in the human colon. After computational improvements and further testing, this system may augment traditional endoscopy when screening the large bowel for occult early malignancies or residual nests of cancer cells following initial oncologic therapy. Though promising, these preliminary results therefore warrant further study. Specifically, future efforts must focus on increasing the image processing speed and further evaluation of the scattering coefficient map and ASI quantification patterns *in vivo*.

## Conclusion

We report the use of swept-source optical coherence tomography and a novel quantitative characteristic to differentiate malignant from normal tissue in nine fresh human colon specimens. Subsurface scattering coefficient maps were generated with a wavelet-based curve fitting method, and angular spectrum indices (ASI) were calculated for each imaged specimen. We found significant qualitative and quantitative differences between normal and malignant tissue. Among this limited sample, we demonstrated that the ASI varies significantly between normal and malignant tissue. While further system optimization and clinical testing are required, we conclude that SS-OCT may provide new diagnostic information when screening for early cancers or surveilling known disease following oncologic therapy. Future work will include system optimization to reduce image processing time, construction of an endoscopic device for further testing, and performance of an appropriately powered *in vivo* study to refine the accuracy of our system.

## Method and Materials

In this section, we describe the colon specimen preparation, the SS-OCT system, the novel wavelet-based-curve-fitting verification, the scattering coefficient mapping generation, the angular spectrum, and the ASI calculation.

### Colon Specimen Preparation

Nine patients undergoing extirpative colonic resection at Washington University School of Medicine were recruited in our initial study. From these patients’ operative specimens, we imaged and processed eight cancers, one pre-malignant polyp, and five representative areas with no gross abnormality. For each image of a specimen, we selected an area 10 mm × 20 mm and processed a region of interest for 3-D mapping of the scattering coefficients. This study was approved by the Institutional Review Board of Washington University School of Medicine, and informed consent was obtained from all patients. All samples were imaged immediately upon resection, prior to fixation in formalin. All methods were performed in accordance with the relevant guidelines and regulations.

### OCT System Setup

The SS-OCT system (details see Supplementary Material) is based on a swept source (HSL-2000, Santec Corp., Japan) with a 1310 nm center wavelength, 110 nm full width at half maximum bandwidth, and 20 kHz scan rate. The interference signal was detected by a balanced detector (Thorlabs PDB450C) and sent to a data acquisition board (ATS9462, Alazartec Technologies Inc). The lateral resolution of the system in air was 10 μm, and the axial resolution was 6 μm. To balance the effects of system signal-to-noise ratio roll-off and Gaussian beam focusing, we performed a calibration test by measuring attenuated mirror signals from different imaging depths.

### Scattering Coefficient Mapping

The scattering coefficient within the colon epithelium layer was calculated by fitting each A-scan with a single attenuation model based on Beer’s law^50–52^: $i(z)\propto \sqrt{\exp [-\,2{\mu }_{t}z]}$, where i(z) is the OCT signal and the factor of 2 accounts for the round-trip attenuation. *μ*_*t*_ = *μ*_*a*_ + *μ*_*s*_ is the total absorption coefficient, which is the summation of the absorption coefficient *μ*_*a*_ and the scattering coefficient *μ*_*s*_. Since in soft tissue *μ*_*a*_ is much less than *μ*_*s*_, the fitted *μ*_*t*_ was used as a good approximation of *μ*_*s*_.

**Figure 8:**
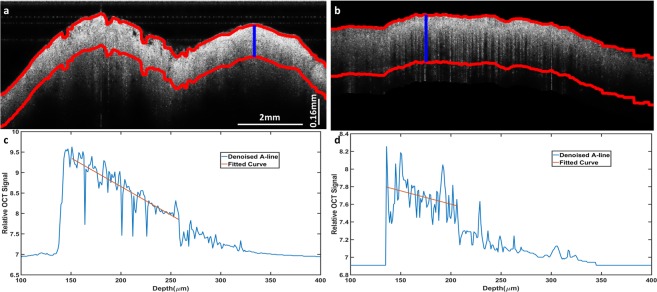
Scattering coefficient fitting. The colon epithelium layer is labeled between two red curves in the de-noised B-scan SS-OCT image (**a**.cancer, **b**.normal). The scattering coefficient of the example A-line (blue) is fitted based on the quantification model, respectively (**c**.cancer, **d**.normal).

We semi-automatically located the colon surface (for details, see Supplementary Materials) and then added a thickness to obtain the epithelium region. The area between the two red curves in Fig. 8a,b identifies the colonic epithelium layer, and the curve fitting for one A-line from the de-noised signal is shown in Fig. 8c,d. All A-lines within a B-scan are fitted. Afterwards we performed this fitting to consecutive B-scans, then generated an *en face* scattering coefficient map of the processed area.

### Data De-noising and Method Verification

In this study, we compared fitted scattering coefficients to the measurement results from data after wavelet analysis, data after applying the nearby average method, and raw data. We used a plug-in, developed by Marco Rossini, in Gimp 2.0 to perform wavelet analysis.

To verify the accuracy of the fitted result, a phantom-based experiment was conducted. Intralipid 20% (manufactured by Fresenius Kabi, Uppsala, Sweden for Baxter Healthcare), and deionized water were used for preparing liquid phantoms with different scattering coefficients. Four different concentrations of intralipid of 1%, 5%, 10%, and 20% were used as liquid phantoms. Then we measured the extinction coefficient, based on *I*(*z*) = *I*(0)exp(−*μ*_*t*_*z*). The experiment schematic is based on Flock *et al*.’s setup^53^. We first measured the light intensity after it was collimated, passed through the cuvette without liquid, passed through two apertures to block forward scattered light and focused by a lens. This intensity was used as *I*(0). Then we filled the hole with liquid phantom and performed the measurement again. This intensity was recorded as *I*(*z*). After substituting these values, we obtained the scattering coefficient. We used the mean of the measurements as our standard. Afterwards we used SS-OCT to scan the phantoms and fitted the scattering coefficients using nearby averaged data, wavelet-analyzed data, and raw data, respectively.

### Angular Spectrum

One new image feature, extracted from the scattering map, is based on the observed differences between the normal and abnormal tissue scattering maps in terms of the spatial distributions. We first cropped the scattering maps to avoid out of focus or hyper-reflection areas. Then 2-D Fourier transform was used to reveal the angular spectrum of these maps (Fig. 6a–d).

A Sobel edge detection was performed in MATLAB to acquire a region with valid frequency information, and the border of the detected edges were fitted to an ellipse using the least squares criterion. The results are shown as blue ellipses in Fig. 6e–h. The ring structure observed in normal tissues represents the periodic crypt pattern. The spatial frequency is derived as

$$f=\sqrt{{f}_{major}^{2}+{f}_{minor}^{2}},$$

where *f*_*major*_ stands for the spatial frequency of the major axis and *f*_*minor*_ stands for the spatial frequency of the minor axis.

To further quantify the ASI, ellipse with a quarter of the area of the blue ellipse was used to depict the signal focused in the center region, which corresponds to the red ellipses in Fig. 6e–h. (See Supplementary Materials for more details). Then, to identify the unique ring structure, we defined the ASI as how much signal is outside the center:

$$\begin{array}{rcl}ASI & = & \frac{all-inner}{all}\\ all & = & integration\,of\,all\,signals\,within\,the\,blue\,ellipses\\ inner & = & integration\,of\,all\,signals\,within\,the\,red\,ellipses\end{array}$$

We then took the average ASI of all cropped areas from one scattering map as the ASI of this map.

### Data analysis

Statistical analysis was performed using MATLAB R2016a. The ASIs of normal and cancerous tissues were compared using student’s t-test, and p < 0.05 was considered statistically significant.

## Supplementary information

The Angular Spectrum of the Scattering Coefficient Map Reveals Subsurface Colorectal Cancer

## Data Availability

The data that support the plots within this paper and other findings of this study are available from the corresponding author upon reasonable request.
