# Supplementary material for: The Angular Spectrum of the Scattering Coefficient Map Reveals Subsurface Colorectal Cancer
Source: Sci Rep. 2019 Feb 28;9:2998. doi: 10.1038/s41598-019-39146-w (PMC6395629; doi:10.1038/s41598-019-39146-w)
Supplement: Supplementary file 1 — The Angular Spectrum of the Scattering Coefficient Map Reveals Subsurface Colorectal Cancer [file 41598_2019_39146_MOESM1_ESM.docx]

**The Angular Spectrum of the Scattering Coefficient Map Reveals Subsurface Colorectal Cancer**

Yifeng Zeng^1,*^, Bin Rao^1,*^, William Chapman Jr^2,*^, Sreyankar Nandy^1^, Rehan Rais^3^, Iván González^3^, Deyali Chatterjee^3^, Matthew Mutch^2^ & Quing Zhu^1,4^

***SS-OCT Schematic.*** The SS-OCT system is based on a swept source (HSL-2000, Santec Corp., Japan) of 1310 nm center wavelength, 110 nm full width at half maximum bandwidth, and 20 kHz scan rate. The interference signal was detected by a balanced detector (Thorlabs PDB450C) and acquired by a data acquisition board (ATS9462, Alazartec Technologies Inc). The schematic is shown in Figure s1.


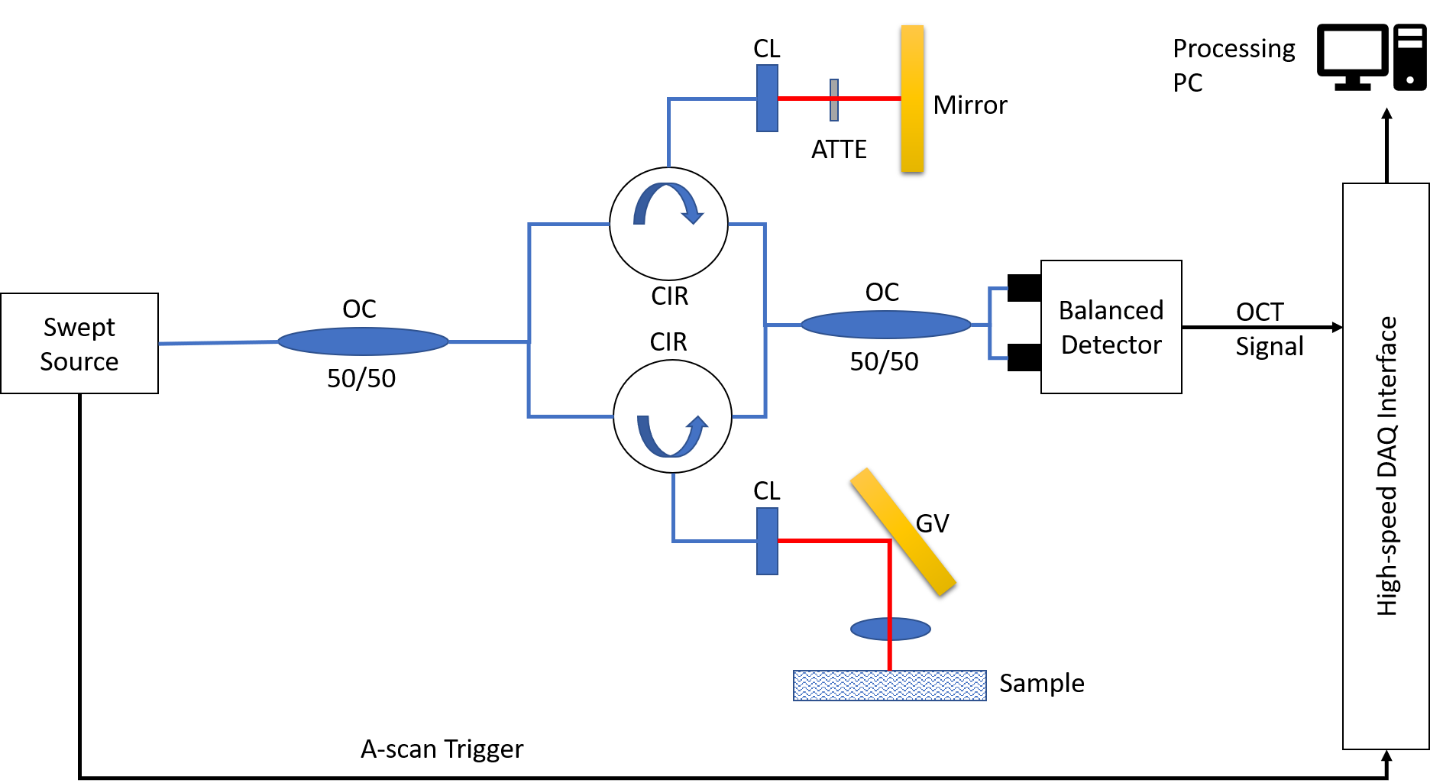


**Figure s1. SS-OCT configuration.** OC: optical coupler; CIR: circulator; CL: collimator; ATTE: attenuator; GV: galvanometer.

^1^ Department of Biomedical Engineering, Washington University, St. Louis, MO. ^2^ Department of Surgery, Section of Colon and Rectal Surgery, Washington University School of Medicine, St. Louis, MO. ^3^Department of Pathology and Immunology, Washington University School of Medicine, St. Louis, MO. ^4^Department of Radiology, Washington University School of Medicine, St. Louis, MO. ^*^These authors contributed equally to this work. Correspondence and requests for materials should be addressed to Y.Z. (email: [yifeng.zeng@wustl.edu](mailto:yifeng.zeng@wustl.edu)) or Q.Z. (email: [zhu.q@wustl.edu](file:///E:\Yifeng%20Zeng\!PhD%20Work\Publications\Journal%20Publication-2-Colon\zhu.q@wustl.edu)).

***Automatic B-scan Surface Delineation.*** We model the surface delineation as an optimization problem. In physics, when travelling between two points, particles tend to follow the route that has the lowest potential energy. Here, we consider the tissue surface as the travelling path and establish a potential function $V(\boldsymbol{l})$ to derive it.

We first treat the SS-OCT image as a n-by-m matrix $I(n,m)$ with each entry represents the OCT signal. Then we use a 1-by-m vector $\boldsymbol{l}$ represents a possible path from the first column to the last column. The ***i***th entry of $\boldsymbol{l}$ is the row number of the path at the ***i***th column and each entry has n possible values in this case. We establish the potential function $V(\boldsymbol{l})$ based on two observations: the detectable sample surface is generally continuous, and the surface has a higher OCT signals than surroundings especially under hyper-reflection situation. The $V(\boldsymbol{l})$ for the ***i***th entry of $\boldsymbol{l}$ is calculated as:

$$V\left( \boldsymbol{l}\left( i \right) \right)=C\left( i \right)+\alpha*Diff\left( i \right)+V\left( \boldsymbol{l}\left( i-1 \right) \right)$$

where $C\left( i \right)$ stands for the continuity, $Diff\left( i \right)$ represents the signal gradient, and $\alpha$ is a custom defined scaling coefficient. $C\left( i \right)$ is defined as:

$$C\left( i \right)=\left| \boldsymbol{l}\left( i \right)-\boldsymbol{l}\left( i-1 \right) \right|$$

and $Diff\left( i \right)$ is calculated by the following equation:

$$Diff\left( i \right)=\sum_{j=\boldsymbol{l}(i)-w}^{\boldsymbol{l}(i)-1} I(j,i)-\sum_{j=\boldsymbol{l}(i)}^{\boldsymbol{l}(i)+w-1} I(j,i)$$

where $w$ is the custom-defined window size to calculate the local gradient. Finally, we can find the surface $\boldsymbol{s}$ by solving the optimization problem:

$$\boldsymbol{s}=\underset{\boldsymbol{l}}{argmin} V\left( \boldsymbol{l} \right)$$

We use dynamic programming to realize this algorithm in MATLAB. In the study, we choose the window size as 15 and $\alpha$ as 0.01 for the best performance. The delineation result is shown in Figure s2.


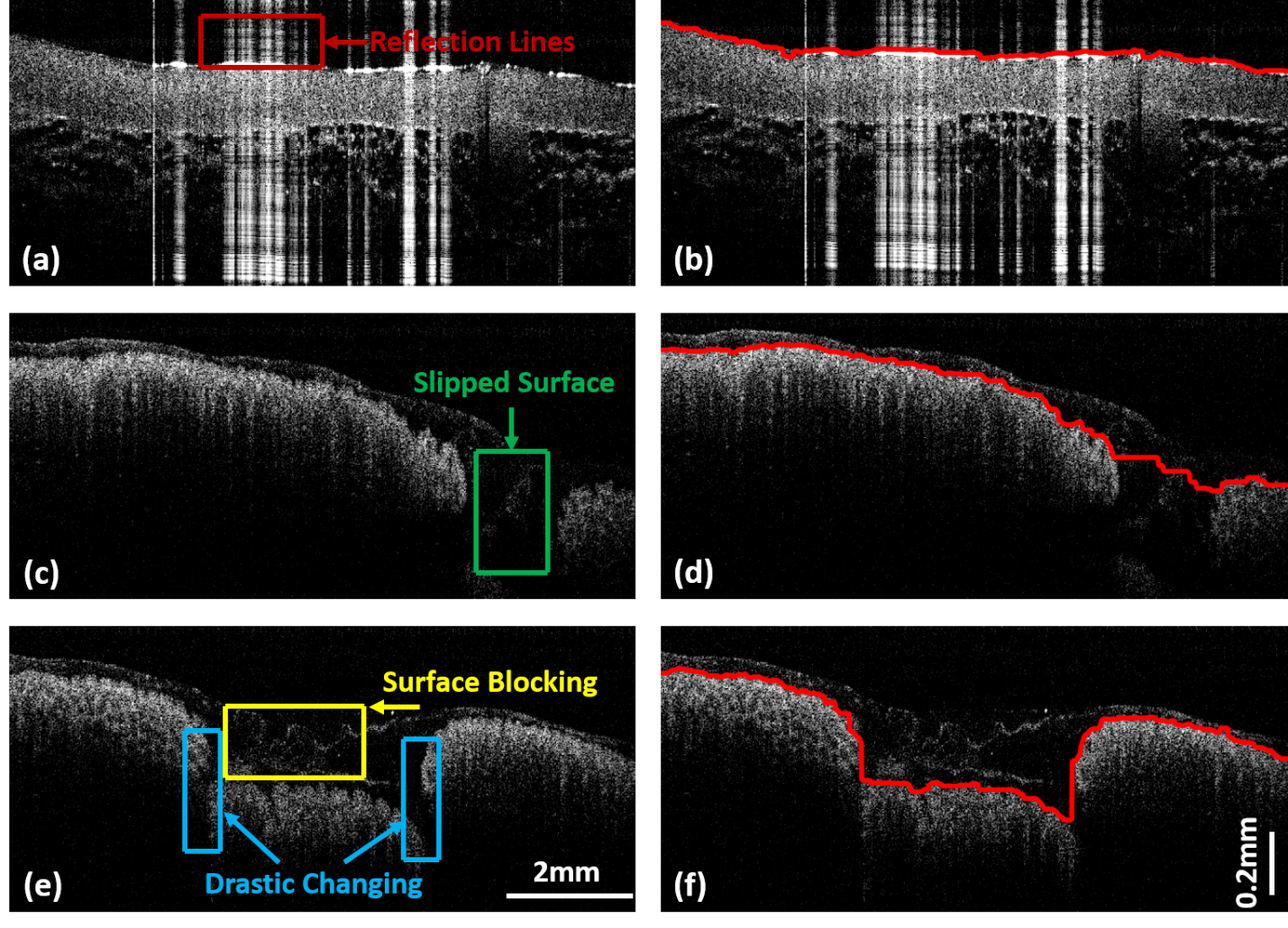


**Figure s2. Surface detection of typical SS-OCT images with challenges.** *Ex vivo* human colon SS-OCT images with hyper-reflection, surface discontinuation, surface blocking, and drastic changing are shown in **(a)**, **(c)**, and **(e)**. **(b)**, **(d)**, and **(f)** show the corresponding surface detection result, respectively, after applying new automatic surface delineation method. Red line depicts the detected surface. All images share the same scale bar.

***Angular Spectrum Ellipse Fitting.***  2-D Fourier transform was applied to cropped scattering maps to generate the angular spectrum (Fig. s3a). A Sobel edge detection was performed in MATLAB to acquire a region with valid frequency information (Fig. s3b), and the border of the detected edges were fitted to an ellipse using the least squares criterion (Fig. s3c blue ellipse). To further separate the ring structure observed in the angular spectrum of normal tissues, we manually generated a red ellipse which has one quarter of area of the blue ellipse (Fig. s3d red ellipse).

**
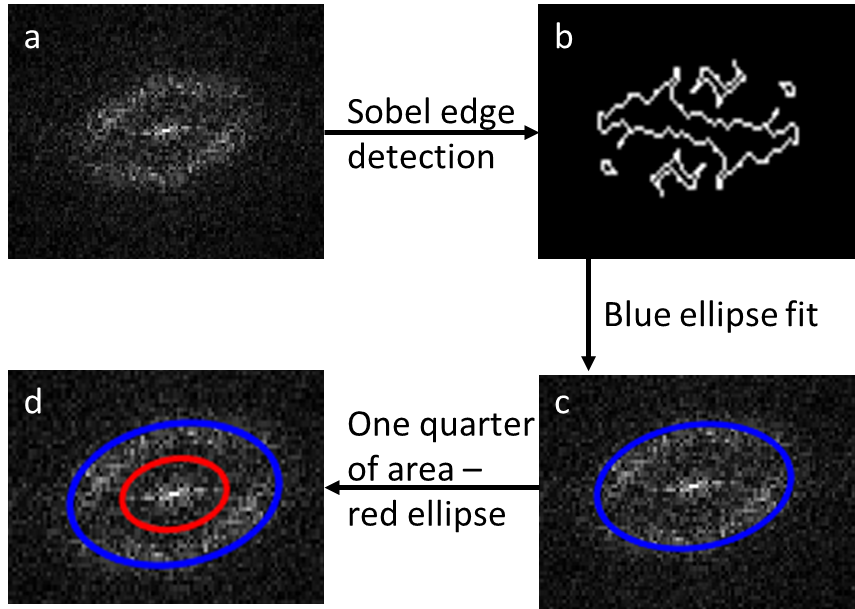
**

**Figure s3. Angular spectrum ellipse fitting. a.** original angular spectrum. **b.** image edge after Sobel edge detection. **c.** blue ellipse fitted based on least squares criterion using the border of the detected edges. **d.** a red ellipse of one quarter of the area of the blue ellipse is drawn to emphasize the ring structure.

**High Scattering Coefficient Area Analysis.** Some highly scattering regions may look heterogeneous in normal scattering coefficient maps due to stronger scattering, e.g., the upper right portion of Fig. 4b. We cropped and visualized this region separately (Fig. s4a) and it shows a periodic pattern similar to normal regions. Moreover, we quantified the ASI of this cropped area and it lies within normal tissue ranges (Fig. s4b).


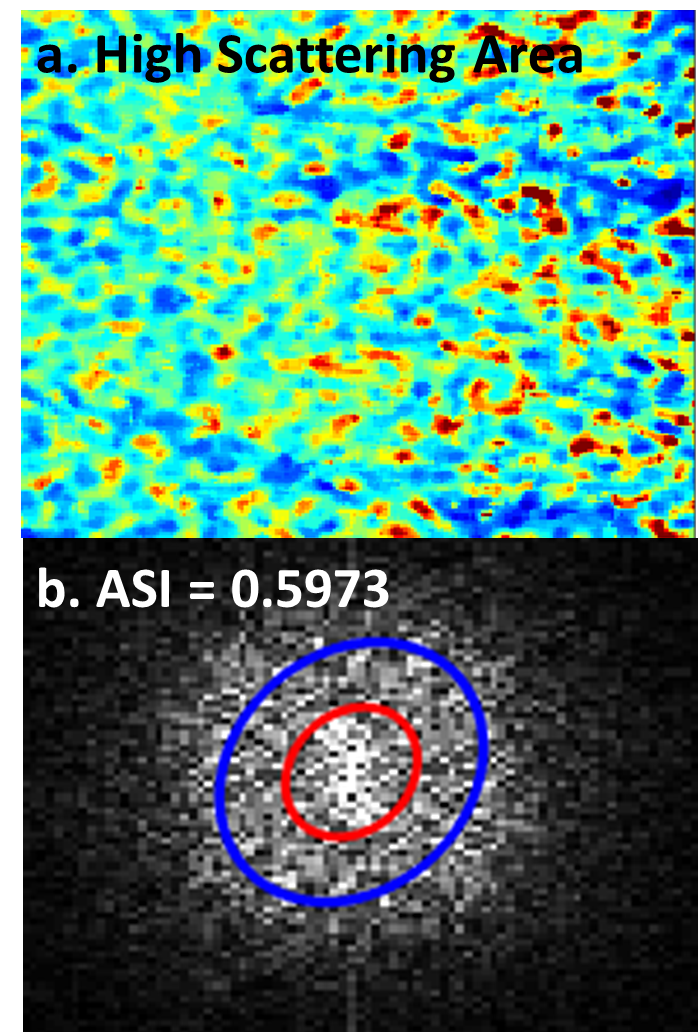


**Figure s4. a.** scattering coefficient map with strong reflector; **b.** angular spectrum of this area. ASI is 0.5973 which is within normal colon range.

**Comparison with depth averaged intensity projection.** We have also compared the direct *en face* OCT images to the scattering coefficient maps. Figure s5 shows the depth-averaged-intensity-projection image and Figure s6 shows the scattering map of the same area. The red box in Figure s5 shows an area that successfully reveals the crypt structure, and the white box in Figure s5 is an area that fails to show this structure. However, in the same areas in Figure s6 (red and white boxes), scattering maps successfully reveal the crypt structure in both areas. We further investigated the angular spectrum index of these two areas. First, the angular spectrum is shown in Figure s7. It can be observed that the angular spectrum of the scattering map has a clearer ring structure. Second, we further quantified the angular spectrum index. The red area in Figure s5 yields a result of 0.511, which appears similar to the angular spectrum of normal tissues; however, the white area has a result of 0.4251, which lies within the range of cancerous tissue. Therefore, using pure *en face* OCT images may lead to misclassification, as demonstrated in this case. One representative B-scan chosen within the white area is shown in Figure s8. We can still visualize the crypt’s gladular structure with B-scan images. However, when the surface is either too thick or has hyper-reflection bands, the dentated structure underneath it will have a very weak signal, which can be overwhelmed in the depth-averaged-intensity-projection map. Since scattering coefficients are used to extract functional information out of OCT images, it will not face this problem and it can visualize the crypt structure well in the scattering coefficient map. To further demonstrate this, we extract the angular spectrum index of the depth-averaged intensity projection maps using the same criteria for the scattering maps. The result is shown in Figure s9. As we can observe, cancer cases and normal cases cannot be well separated. There are some overlaps and the p-value is 0.08 which is considered not statistically significant. Thus, we believe the method based on scattering coefficient fitting provides more accurate results.


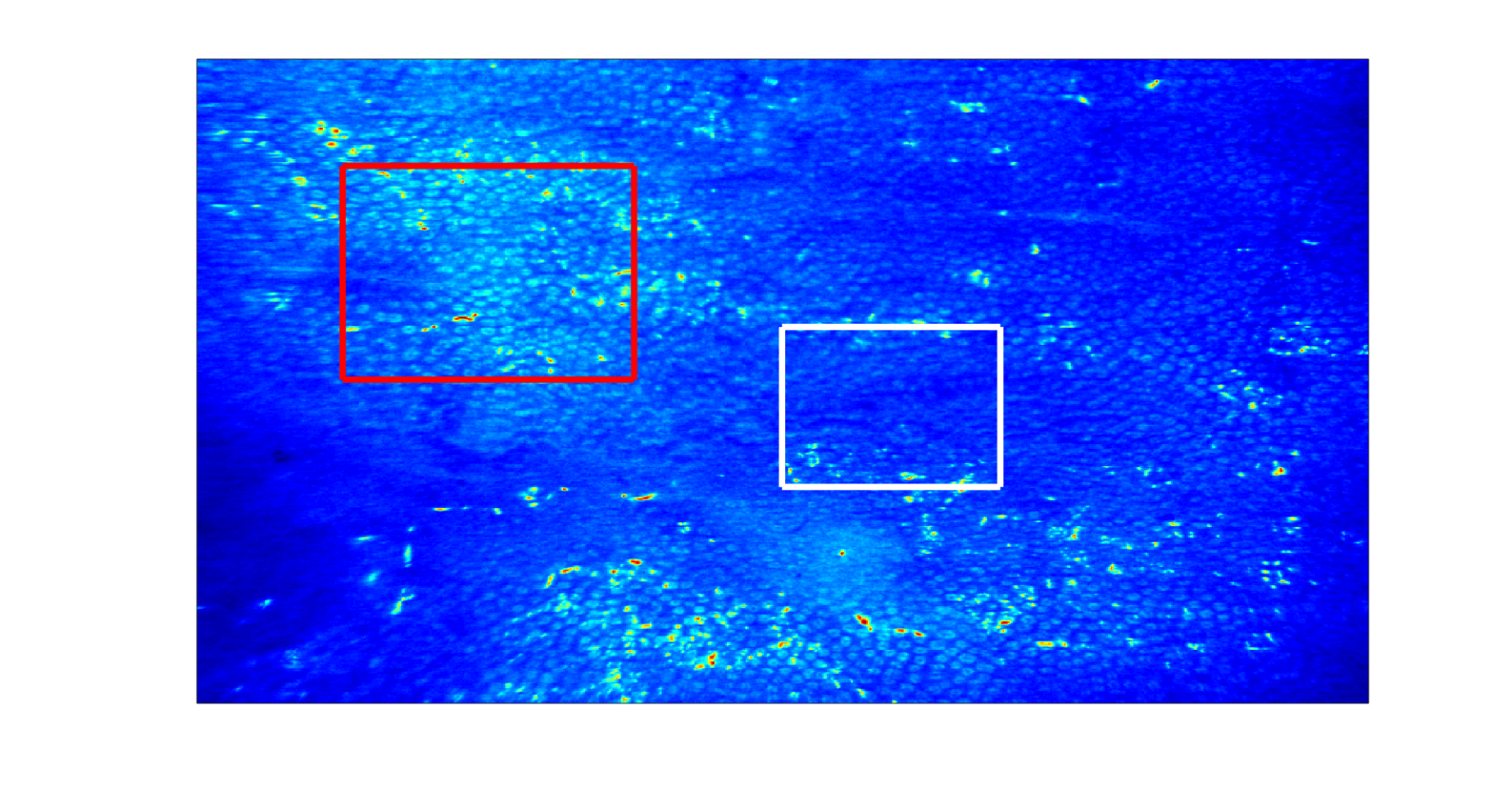


**Figure s5. OCT depth-averaged-intensity-projection image.** Red box represents an area that reveal the crypt pattern. White box shows an area that fail to reveal the crypt pattern.


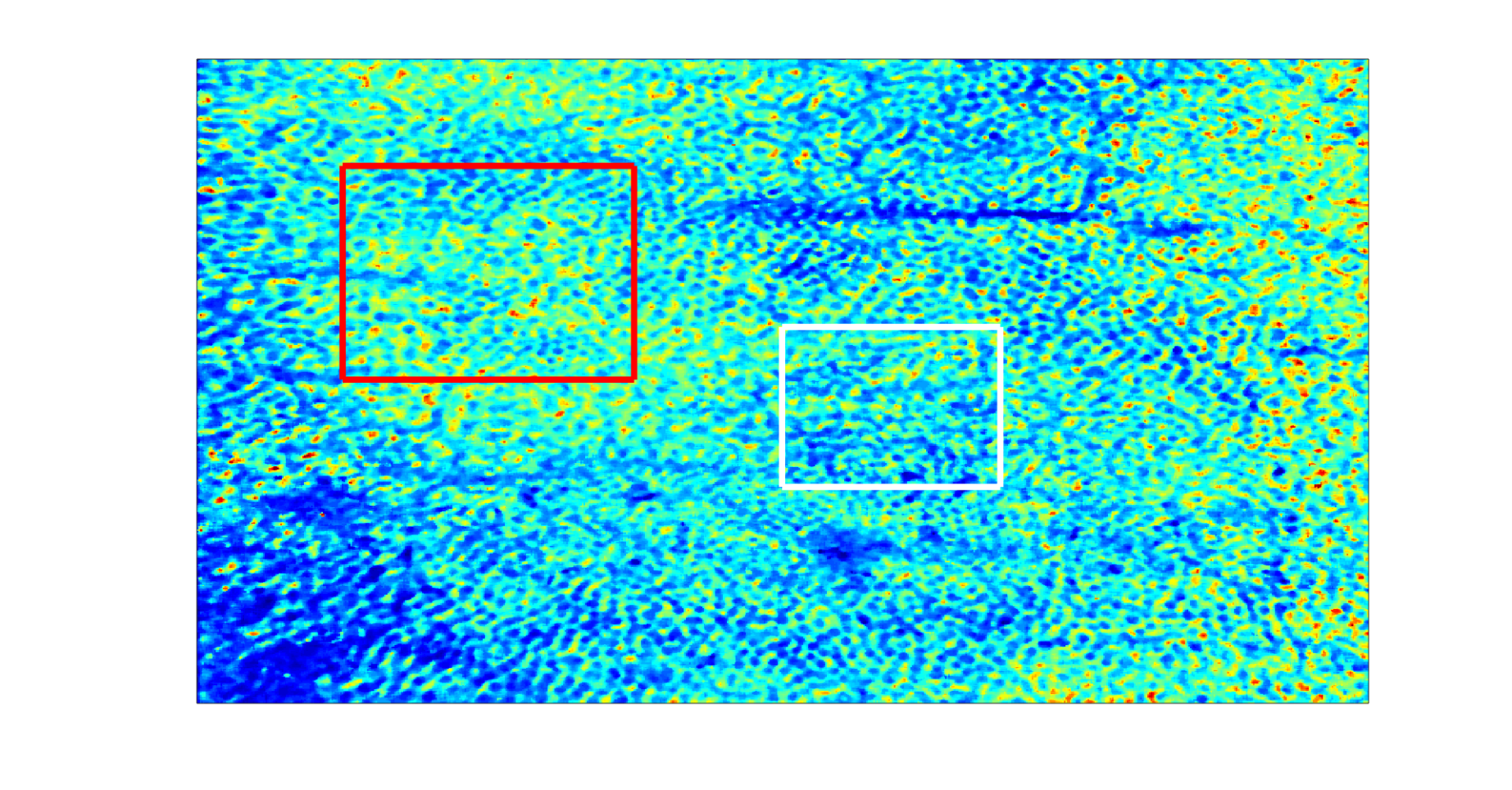


**Figure s6. OCT scattering map of the same area of figure s5.** Red box and white box are the same position in figure s5. Both of them reveal the crypt structure successfully.


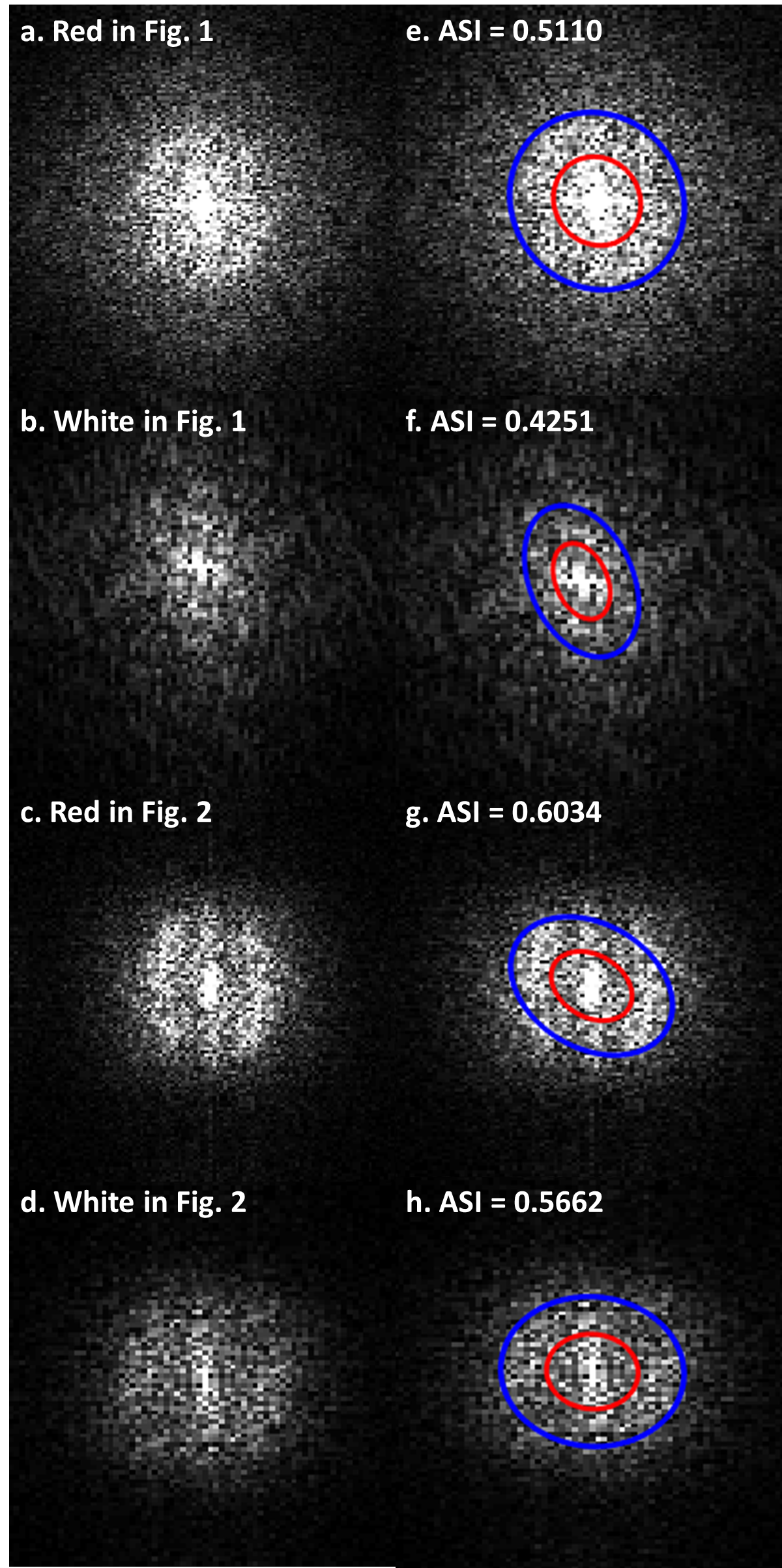


**Figure s7. Angular spectrum of red and white areas in Fig. s5 and Fig. s6. a.-d.** angular spectrum. **e.-h.** angular spectrum with fitted ellipses. It can be observed that the scattering coefficient map (Fig. s6) has a clearer ring structure.


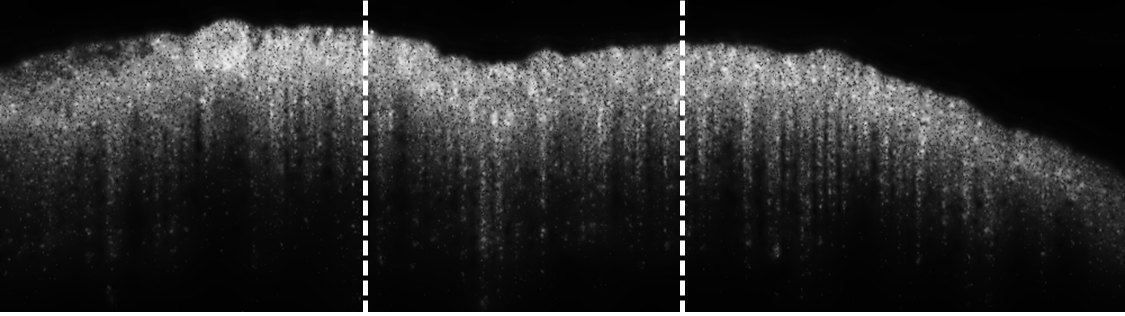


**Figure s8. Representative B-scan within the white box in Fig. s5.** Two white dashed lines correspond to the boundary in Fig. s5. The dentate structure can still be visualized.


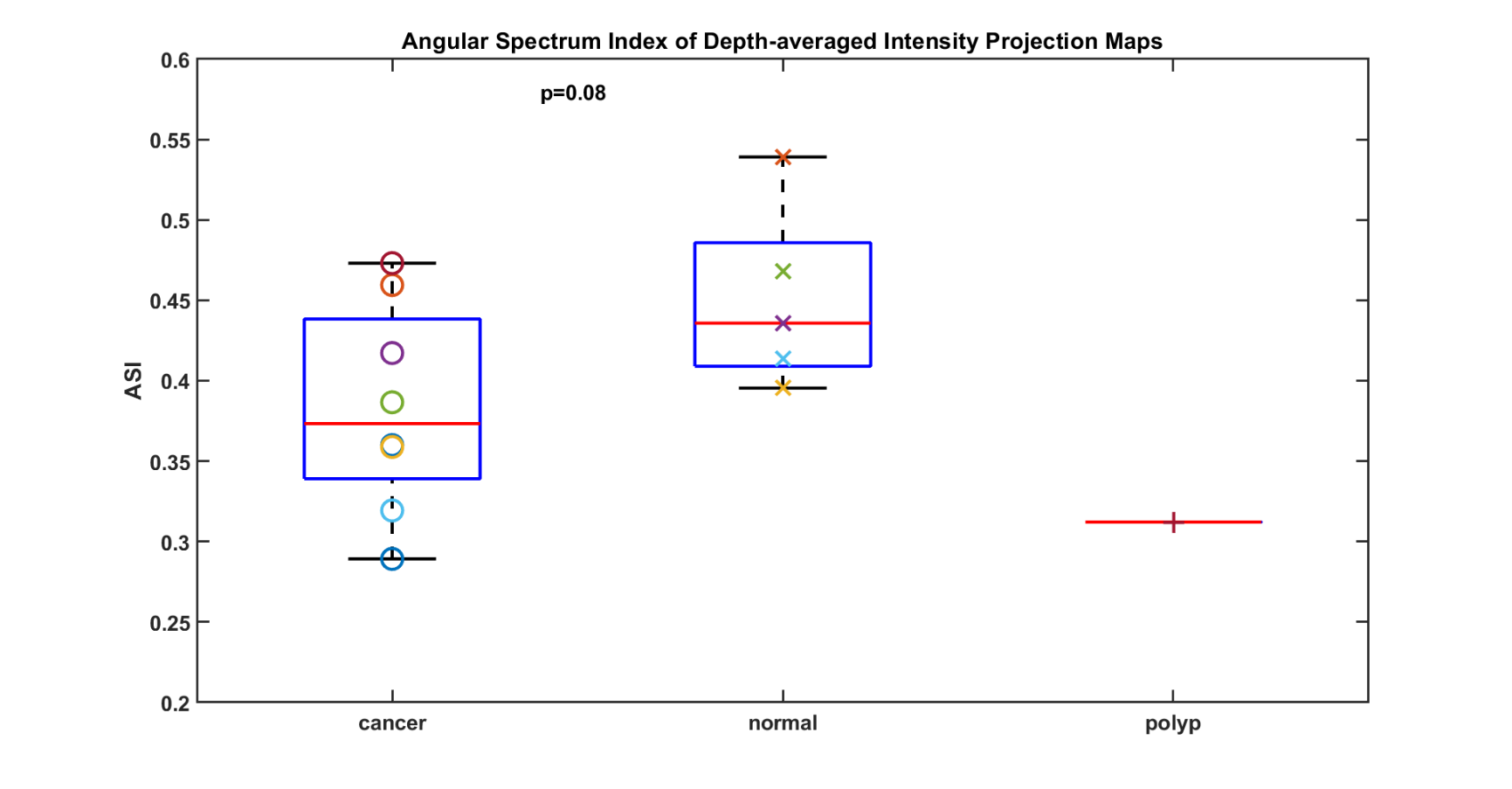


**Figure s9. Angular Spectrum Index of Depth-averaged Intensity Projection Maps.** Cancer cases and normal cases cannot be well separated. There are some overlaps and the p-value is 0.08 which is not statistically significant.
